# Incorporating oral PrEP into standard prevention services for South African women: a nested interrupted time-series study

**DOI:** 10.1016/S2352-3018(21)00048-5

**Published:** 2021-06-11

**Authors:** Deborah Donnell, Ivana Beesham, Julia D Welch, Renee Heffron, Melanie Pleaner, Lara Kidoguchi, Thesla Palanee-Phillips, Khatija Ahmed, Deborah Baron, Elizabeth A Bukusi, Cheryl Louw, Timothy D Mastro, Jennifer Smit, Joanne R Batting, Mookho Malahleha, Veronique C Bailey, Mags Beksinska, Helen Rees, Jared M Baeten, ECHO Trial Consortium, Peter B Gichangi, Kate B Heller, Nomthandazo Mbandazayo, Charles S Morrison, Kavita Nanda, Caitlin W Scoville, Kathleen Shears, Petrus S Steyn, Douglas Taylor, Katherine K Thomas, James Kiarie, Jessica Justman, Zelda Nhlabatsi, Linda-Gail Bekker, Gonasagrie Nair, G Justus Hofmeyr, Mandisa Singata-Madliki, Raesibe Agnes Pearl Selepe, Sydney Sibiya, Margaret Phiri Kasaro, Jeffrey Stringer, Nelly R Mugo

**Affiliations:** a Fred Hutchinson Cancer Research Center, Vaccine and Infectious Disease Division, Seattle, WA, USA; b MatCH Research Unit (MRU), Department of Obstetrics and Gynaecology, Faculty of Health Sciences, University of the Witwatersrand, Durban, South Africa; c FHI 360, Durham, NC, USA; d University of Washington, Seattle, WA, USA; e Wits Reproductive Health and HIV Institute, Faculty of Health Sciences, University of the Witwatersrand, Johannesburg, South Africa; f Setshaba Research Centre, Tshwane, South Africa; g Center for Microbiology Research, Kenya Medical Research Institute (KEMRI), Nairobi, Kenya; h Madibeng Centre for Research, Brits, South Africa; i Effective Care Research Unit (ECRU), Universities of the Witwatersrand, Fort Hare and Eastern Cape Department of Health, East London, South Africa; j Department of Family Medicine, Faculty of Health Sciences, University of Pretoria, Pretoria, South Africa; k Gilead Sciences, Foster City, CA, USA

## Abstract

**Background:**

As oral pre-exposure prophylaxis (PrEP) becomes the standard of prevention globally, its potential effect on HIV incidence in clinical trials of new prevention interventions is unknown, particularly for trials among women. In a trial measuring HIV incidence in African women, oral PrEP was incorporated into the standard of prevention in the trial's last year. We assessed the effect of on-site access to PrEP on HIV incidence in this natural experiment.

**Methods:**

We did a nested interrupted time-series study using data from the ECHO trial. At 12 sites in four countries (Eswatini, Kenya, South Africa, and Zambia), women (aged 16–35 years) were randomly assigned to receive one of three contraceptives between Dec 14, 2015, and Sept 12, 2017, and followed up quarterly for up to 18 months to determine the effect of contraceptive method on HIV acquisition. Women were eligible if they wanted long-acting contraception, were medically qualified to receive study contraceptives, and had not used any of the study contraceptives in the past 6 months. The present analyses are limited to nine South African sites where on-site access to oral PrEP was implemented between March 13 and June 12, 2018. Using an interrupted time-series design, we compared HIV incidence before versus after PrEP access, limited to quarterly study visits at which on-site PrEP access was available to at least some participants and, in a sensitivity analysis, to the 180 days before and after access. The outcome was incident HIV infection, detected using two rapid HIV tests done in parallel for each participant at every scheduled follow-up visit. This study is registered on ClinicalTrials.gov, NCT02550067.

**Findings:**

2124 women were followed up after on-site PrEP access began, of whom 543 (26%) reported PrEP use. A total of 12 HIV seroconversions were observed in 556 person-years (incidence 2·16%) after on-site PrEP access, compared with 133 HIV seroconversions in 2860 person-years (4·65%) before PrEP access (adjusted incidence rate ratio [IRR] 0·45, 95% CI 0·25–0·82, p=0·0085). Similar results were also observed when limiting the analysis to 180 days before versus after PrEP access. A total of 46 HIV seroconversions were observed in 919 person-years within 180 days before PrEP access, compared with 11 seroconversions in 481 person-years in the 180 days following PrEP access (incidence 5·00 *vs* 2·29 per 100 person-years; IRR 0·43, 95% CI 0·22–0·88, p=0·012).

**Interpretation:**

On-site access to PrEP as part of standard of prevention in a clinical trial among women in South Africa was associated with halving HIV incidence, when approximately a quarter of women started PrEP. Providing access to on-site PrEP could decrease incidence in HIV prevention trials. These data are also among the first to show in any setting that access to PrEP is associated with decreased HIV acquisition among South African women.

**Funding:**

Bill & Melinda Gates Foundation, United States Agency for International Development, President's Emergency Plan for AIDS Relief, the Swedish International Development Cooperation Agency, South African Medical Research Council, and United Nations Population Fund.

## Introduction

In 2015, WHO recommended pre-exposure prophylaxis (PrEP) as a HIV prevention option for populations worldwide at high risk for acquiring HIV (defined in the recommendation guidance as having an HIV incidence of >3·0 per 100 person-years).^1^, ^2^ PrEP has subsequently been incorporated into national HIV prevention policies and delivered in demonstration and implementation projects to diverse populations globally.^3^ However, few projects have assessed the impact of PrEP access on HIV incidence, particularly for African women using PrEP. PrEP studies in women have been completed in South Africa, Kenya, Uganda, Zimbabwe, and Zambia, in which women had low PrEP adherence^4^, ^5^ and women-focused HIV prevention efficacy and implementation projects have found variable uptake and often low continuation of PrEP.^6^, ^7^, ^8^, ^9^

Clinical trials of novel HIV prevention products, such as new PrEP drugs or vaccines, routinely provide a comprehensive suite of prevention interventions—such as risk-reduction counselling, free condoms, treatment of sexually transmitted infections, partner HIV testing, and antiretroviral therapy referral—collectively referred to as standard of prevention. Since 2015, trials have navigated a complex path between the evolving process of implementation and access based on in-country PrEP guidelines and decisions about study-specific access to PrEP for trial participants.^10^, ^11^, ^12^ Ethical guidance from WHO and UNAIDS has declared that prevention trials should offer an optimised standard of prevention package, incorporating new prevention strategies as they show effectiveness and are incorporated into national policies.^13^

**Research in context**

**Evidence before this study**

We searched PubMed (n=69) and Web of Science (n=134) for trials assessing HIV pre-exposure prophylaxis (PrEP) in women from database inception to Feb 21, 2021, in English. We used the search terms “HIV incidence” AND (“PrEP” OR “pre-exposure prophylaxis”) AND “women” NOT “transgender”. We restricted the search to articles and conference abstracts. Randomised clinical trials of tenofovir disoproxil fumarate and emtricitabine as PrEP for HIV prevention done in women in Africa had effectiveness that varied from highly effective to ineffective. Following the 2015 WHO recommendation for PrEP use in women, PrEP demonstration and implementation projects in Africa have shown varying uptake and challenges with adherence. Very few studies have assessed the effect of PrEP on HIV incidence in African women.

**Added value of this study**

This study assessed the direct effect on HIV incidence of offering integrated on-site access to PrEP in clinical trial participants as part of the standard of prevention. With a quarter of the women choosing to use PrEP, HIV incidence was reduced by 50%, adding effectiveness evidence to the known efficacy of PrEP in women.

**Implications of all the available evidence**

Offering on-site access to PrEP as part of the standard of prevention reduced HIV incidence in clinical trial participants in South Africa. Future clinical trials should anticipate lower HIV incidence rates when PrEP is integrated into standard prevention services.

The ECHO trial^14^ was a randomised trial done to evaluate whether the risk of HIV acquisition differed substantially for women using one of three long-acting reversible contraceptives. Use of PrEP was explicitly permitted in the protocol, which stated that PrEP could be provided on-site as guidelines changed and participants became PrEP eligible. In November, 2017, the South African Medical Research Council convened a stakeholder summit to discuss standard of prevention in clinical trials; the summit recommended the provision of PrEP to clinical trial participants at South African research sites in HIV prevention clinical trials.^15^ At the Eswatini, Kenya, and Zambia sites, PrEP continued to be accessed through self or site referral to off-site facilities that provided PrEP, such as national programmes and PrEP demonstration projects, as had occurred from the study outset. At the nine South African sites, ECHO expanded PrEP access by implementing on-site access to daily tenofovir disoproxil fumarate and emtricitabine, following community consultation, regulatory body notification, staff training, and operational readiness preparation.^16^ The change to PrEP provided on-site by study staff began at all nine sites in the last year of the study, between March and June, 2018, and the risk of HIV acquisition continued to be assessed. This natural experiment offered an opportunity to assess whether on-site access to PrEP at the South African clinical sites reduced HIV incidence.

## Methods

### Study design and participants

We did a nested interrupted time-series study using data from the ECHO trial. Women (aged 16–35 years) from 12 sites (freestanding research centres, university-affiliated research centres, and clinical sites providing reproductive health services) in four countries (Eswatini, Kenya, South Africa, and Zambia) who were HIV negative, seeking long-acting reversible contraception, and willing to be randomly assigned to copper intra-uterine device, intramuscular depot medroxyprogesterone acetate, or levonorgestrel implant were enrolled in the ECHO trial between Dec 14, 2015, and Sept 12, 2017. They were followed up for up to 18 months, with follow-up completing on Oct 31, 2018. Women who were not medically eligible for any contraceptives or did not use any of the contraceptives in the past 6 months were excluded. The present analyses are limited to the South African sites (nine of 12 trial sites, accounting for 74% of enrolments into the trial) because PrEP access was implemented on-site by study staff with a documented approval date to begin on-site access; in the other three sites, PrEP was offered off-site only through national programmes and demonstration and implementation projects, with a more gradual change in access for study participants that mirrored availability for the general population in Eswatini, Kenya, and Zambia. Before on-site access, PrEP was similarly available through demonstration and implementation projects at several South African public health facilities external to the study sites.

Global and site-specific community advisory groups provided ongoing input into trial conduct, including PrEP delivery, and ethics review committees approved the study protocol. All participants provided written informed consent and were counselled about their rights as research participants.

### Procedures

The study outcome was incident HIV infection, detected using two rapid HIV tests done in parallel for each participant at every scheduled follow-up visit.

Follow-up visits occurred every 3 months, and included HIV testing, using a standard algorithm.^14^ All women were offered a standard HIV prevention package at all visits, which included HIV testing, HIV risk reduction counselling (including information about PrEP), condom provision, and syndromic management of symptomatic sexually transmitted infections.

The trial protocol permitted the use of PrEP by all participants and provision on-site as it became part of the country's standard of prevention. PrEP was introduced in South Africa in 2016, prioritised for sex workers, and was expanded in 2017 to include additional at-risk groups including young women, adolescent girls (aged 10–24 years), and men who have sex with men. After the PrEP access resolution at the Nov 6, 2017, South African Medical Research Council summit, the ECHO team implemented access to PrEP in the nine South African trial sites, accompanied by staff training aligned with South Africa Department of Health PrEP technical working group mandates, as previously described.^16^ Briefly, discussion and input from local ethics committees and community stakeholders was included in the decision to provide PrEP on-site and offer it voluntarily to study participants. Between January, 2018, and March, 2018, study site staff were trained, using a standardised training adapted from the South African National Department of Health PrEP guidelines.^17^ Training included the importance of the participant understanding her own risk profile, communication about adherence to PrEP, and information about post-study access. PrEP was offered by study staff, aligned with ECHO visits. Women who initiated PrEP received adherence counselling by study clinicians and counsellors at follow-up visits, and any concerns or side-effects were addressed and managed. Objective markers of adherence such as pill counts and drug detection in biological samples were not done.

The start date for on-site PrEP was defined as the specific date the site was approved to provide PrEP by the study leadership committee: the first approval was on March 13, 2018, with all sites initiated by June 12, 2018. Once on-site access began, study staff offered PrEP to study participants as part of the HIV prevention package at ECHO follow-up study visits. PrEP access for a participant was defined at their first study visit with an HIV test following start of on-site PrEP. Throughout follow-up, PrEP use was ascertained by the question “since the last visit, has the participant used oral or topical PrEP against HIV?”; data on adherence to PrEP or source of PrEP prescription (ie, on-site or off-site provider) were not collected in the database. After on-site PrEP access began, all women still in active follow-up were offered PrEP at each study visit. At the end of the study, women who wanted to continue PrEP were given a 3-month supply and referred to off-site PrEP providers in the community.

### Statistical analysis

This analysis compared HIV incidence after implementation of on-site PrEP access with previous off-site access, to determine the effect of PrEP access, for all participants remaining in follow-up (ie, not only those who used PrEP), on incident HIV. Participants' first PrEP access visit was their first scheduled study visit (which included a HIV test) following the site-specific on-site PrEP start date. Women using PrEP before the site start date (ie, those who obtained PrEP from an external programme) were included as not having on-site PrEP access for those visits, despite having initiated PrEP.

PrEP access, because it began on a fixed date at each site, was confounded by calendar time, and two analytical approaches were implemented to limit that confounding. First, we included only study visit intervals for which at least some participants had on-site access to PrEP. This meant that only time from study months 12, 15, and 18 were included, as at these visits some participants had access (later enrollees) and some did not (earlier enrollees). Study visits were included for which at least 20 participants had access to PrEP at the site. In a sensitivity analysis, we used an interrupted time-series quasi-experimental design comparing incidence in calendar time in the 180 days before any on-site PrEP access began at the study site and 180 days immediately after access began. In this comparison, intervals that included the transition to PrEP access (ie, contained the PrEP access date) were excluded so that in the intervals before, all women were known to have no site access to PrEP, and in intervals after, all women were known to have had site-access to PrEP.

HIV incidence was calculated as the number of incident HIV infections divided by person-years observed between HIV tests. Poisson regression with generalised estimating equations (GEE) using robust standard errors was used to assess incidence rate ratios (IRRs) for the time-varying covariate PrEP access, adjusted for site and randomised contraceptive group. Fully adjusted analysis additionally included baseline and visit-specific factors with known association with risk for HIV acquisition: women aged younger than 24 years (*vs* those aged ≥24 years), women reporting their partner had other partners (baseline and follow-up), condomless sex (baseline and follow-up), and any new partner (follow-up only).

We did several exploratory analyses to assess whether our study findings simply reflected an ecological trend in HIV incidence. First, we excluded all time after PrEP access, then repeated the analysis methods (poisson regression with GEE) to estimate an IRR based on a fake PrEP access date between 80 weeks and 20 weeks before the true PrEP access date. Second, we directly report the observed incidence rates over study follow-up before PrEP access. Finally, to assess whether the study findings reflected a change in risk in the cohort over the enrolment period, as only later enrolled participants had on-site PrEP access, we compared HIV incidence in the first 12 months of study participation (before on-site PrEP access) for women with and without on-site PrEP access (defined by whether date of enrolment implied the participant remained in follow-up when on-site PrEP was initiated). The analysis used Cox proportional hazards, stratified by site. All analyses were done in R (version 3.6). This study is registered on ClinicalTrials.gov, NCT02550067.

### Role of the funding source

The funder of the study had no role in study design, data collection, data analysis, data interpretation, or writing of the report.

## Results

**Figure 1:**
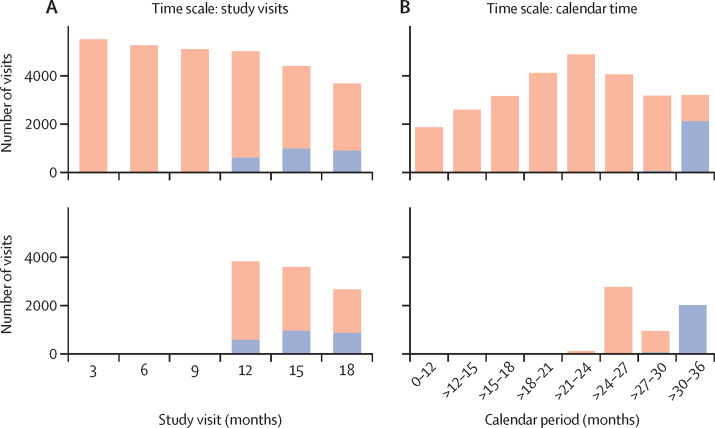
Study visits included in the analysis, compared with total study visits Number of visits with each type of PrEP access are shown for two different time scales. (A) Study visits (months since participant enrolment). (B) Calendar time (month since first participant enrolled). Distribution of visits with PrEP access through the national standard of care (orange) or on-site implementation (blue) are shown. The upper plots show all study visits and the lower plots show study visits that are included in the analysis. For the study visit time scale, study visits were included if both types of access occurred; for the calendar time scale, intervals within 180 days entirely before and after on-site PrEP access are included (intervals including the transition to PrEP access are excluded). PrEP=pre-exposure prophylaxis.

2124 women contributed to follow-up time after on-site PrEP access began; five women were excluded as a result of the inclusions requirement that at least 20 participants at that site and study visit had access to PrEP. Their median age was 23 years (IQR 20–27; table 1) and 64% had a baseline VOICE risk score of 5 or more.^18^ Approximately 50% of the women contributed study time both before and after PrEP access (a profile of study time included compared with all study time for the two methods is shown in figure 1). Sexual risk behaviours reported at visits before and after on-site access were very similar: in 6% of visits, women reported more than one sexual partner; about 10% reported knowing their partner has other partners; sex without a condom was reported in about 65% of visits; and new sexual partners in 5% of visits.

**Table 1:** Description of follow-up time, including demographics and HIV sexual risk behaviours

		**Study visits with PrEP access**		**180 days before and after PrEP access**	
		Before access	After access	Before access	After access
Number of women		4582	2119	3675	2032
Person-years		2860	556	919	481
Baseline VOICE risk score*					
	0–4	1633/4582 (36%)	776/2119 (37%)	1336/3675 (36%)	751/2032 (37%)
	5–9	2931/4582 (64%)	1336/2119 (63%)	2326/3675 (64%)	1274/2032 (63%)
Median age, years		23 (20–26)	23 (21–27)	23 (20–27)	23 (21–27)
Number of visits		10 278	2446	3774	2160
Study month					
	6 months	..	..	51/3774 (14%)	..
	9 months	..	..	797/3774 (21%)	13/2160 (1%)
	12 months	4063/10 278 (40%)	593/2446 (24%)	735/3774 (19%)	585/2160 (27%)
	15 months	3432/10 278 (33%)	967/2446 (40%)	777/3774 (21%)	835/2160 (39%)
	18 months	2783/10 278 (27%)	886/2446 (36%)	955/3774 (25%)	727/2160 (34%)
Number of sexual partners					
	0	278/10 278 (3%)	77/2446 (3%)	86/3774 (2%)	66/2160 (3%)
	1	9366/10 278 (91%)	2236/2446 (91%)	3463/3774 (92%)	1972/2160 (91%)
	2	630/10 278 (6%)	131/2446 (5%)	225/3774 (6%)	122/2160 (6%)
Partner has other sexual partners					
	No	3336/10 278 (32%)	812/2446 (33%)	1216/3774 (32%)	711/2160 (33%)
	Yes	1003/10 278 (10%)	212/2446 (9%)	398/3774 (11%)	186/2160 (9%)
	Do not know	5935/10 278 (58%)	1420/2446 (58%)	2160/3774 (57%)	1263/2160 (58%)
Ever initiated PrEP at or before this visit		270/10 278 (3%)†	612/2446 (25%)	17/3774 (0%)	536/2160 (25%)
Reported any unprotected sex†		6667/10 278 (65%)	1628/2446 (67%)	2459/3774 (65%)	1434/2160 (66%)
New partner		544/10 278 (5%)	112/2446 (5%)	196/3774 (5%)	105/2160 (5%)

Data are n, n (%), or median (IQR). PrEP=pre-exposure prophylaxis.

* VOICE risk score has been validated as a predictor of HIV incidence in African women and includes the following factors: age <25 years; unmarried or not living with partner; partner does not provide financial or material support; primary partner has other partners (yes or do not know); alcohol use in the past 3 months; or having a curable sexually transmitted infection. VOICE risk score was measured at baseline and is a characteristic of the woman at enrollment. All other covariates were assessed at every visit.

† For 227 (88%) of these women, their PrEP initiation date was between ECHO quarterly visits and after initiation of on-site PrEP access. These intervals with partial PrEP access are included in the before access data.

Trial sites began offering on-site PrEP beginning on March 13, 2018, with all sites implementing PrEP by June 12, 2018. Among the 3756 participants still in follow-up on March 1, 2018, a total of 27 (<1%) women had initiated PrEP. By July 1, 2018, 313 (8·3%) women had initiated PrEP, and by the study's conclusion in October, 2018, 542 (26%) of 2119 women who had site access to PrEP had initiated PrEP. A total of 133 HIV seroconversions were observed in 2860 person-years (4·65 per 100 person-years) before PrEP access, compared with 12 HIV seroconversions in 556 person-years (incidence 2·16 per 100 person-years) after PrEP access (adjusted IRR 0·45, 95% CI 0·25–0·82, p=0·0085; table 2). 258 women had initiated PrEP before their first PrEP access visit (ie, their scheduled ECHO visit); however, 227 (88%) of those had a PrEP start date after the date on-site PrEP access began and probably obtained PrEP from the study site.

**Table 2:** Effect of on-site PrEP access on HIV incidence

	**Number of infections/person-years**	**Incidence**	Adjusted incidence rate ratio*(95% CI)	**p value**	**Adjusted incidence rate ratio**†**(95% CI)**	**p value**
**Including study visits with on-site PrEP access**						
Before access	133/2860	4·65%	..	..	..	..
After access	12/556	2·16%	0·45 (0·25–0·81)	0·0076	0·45 (0·25–0·82)	0·0085
**180 days before versus 180 days after on-site PrEP access**						
Before access	46/919	5·00%	..	..	..	..
After access	11/481	2·29%	0·44 (0·23–0·85)	0·015	0·43 (0·22–0·83)	0·012

Data are n/person-years, incidence, rate ratio (95% CI), or p value. PrEP=pre-exposure prophylaxis.

* Adjusted for study site and randomisation group.

† Adjusted for study site, randomisation arm, age <24 years, woman reporting her partner had other partners (baseline and follow-up), any unprotected sex (baseline and follow-up), and any new partner (follow-up only).

A total of 46 HIV seroconversions were observed in 919 person-years within 180 days before PrEP access, compared with 11 seroconversions in 481 person-years in the 180 days following PrEP access (incidence 5·00 *vs* 2·29 per 100 person-years; adjusted IRR 0·43, 95% CI 0·22–0·83, p=0·012). Only 17 visits with PrEP use were included in the 180 days before PrEP access, compared with 536 visits in the 180 days after on-site access.

**Figure 2:**
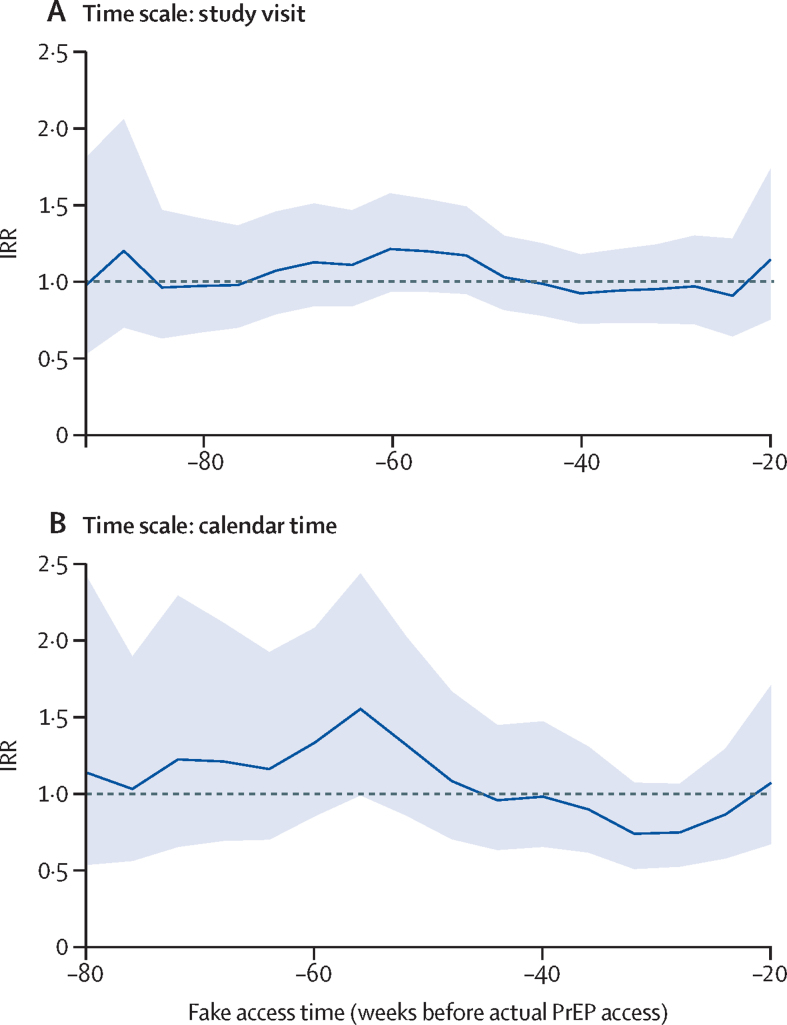
IRR comparing HIV incidence before and after fake dates of on-site PrEP access Change in HIV incidence for a fake access date is compared using two different time scales, as for the analysis of true date of on-site access. (A) Study visit showing a comparison within the same study visits for women with and without access to PrEP. (B) Calendar time showing a comparison close to the same calendar time for women with and without access. IRR=incidence rate ratio. PrEP=pre-exposure prophylaxis.

HIV incidence trends, independent of PrEP access, remained close to 5·0% throughout follow-up (appendix p 1). If fake PrEP access dates were set, earlier than the true access dates, and applied to the two analytical approaches previously mentioned (excluding time with on-site access), the IRR was close to 1 for a range of dates (figure 2). For women who enrolled earlier into the ECHO trial (ie, whose follow-up was complete before PrEP access began), HIV incidence in their first 12 months was 5·19 per 100 person-years, whereas among later-enrolled women who eventually had on-site PrEP access, incidence in their first 12 months (excluding any PrEP access time) was 4·50 per 100 person-years; these rates were not statistically different (hazard ratio 0·85, 95% CI 0·66–1·1, p=0·22).

## Discussion

In the ECHO trial, on-site provision of PrEP at no cost to participants was introduced part way through the clinical trial, providing an opportunity to assess HIV incidence in the population after access to PrEP versus before access to PrEP. Notably, our analysis did not limit assessment of HIV incidence to just those women who initiated PrEP, instead assessing incidence in the entire study cohort after on-site access. Our analyses showed a decrease of about 50% in HIV incidence after on-site access to PrEP, even with a modest 26% of women initiating PrEP. These findings are of direct importance to future studies of HIV prevention that will incorporate PrEP as part of standard of prevention. Moreover, the findings show that, among South African women provided access to quality PrEP services, HIV incidence declined substantially.

Other studies have also reported low HIV incidence in women with access to PrEP. HPTN 082^6^ was a demonstration study of PrEP among adolescent girls and young women at two sites in South Africa and one in Zimbabwe; a low HIV incidence of 1·0 per 100 person-years (95% CI 0·3–2·5) was observed, despite only 25% of women with biomarkers suggesting sustained adherence to PrEP. Further work in this cohort found higher adherence to PrEP was correlated with known risk factors for HIV, suggesting that women at higher risk might choose to accept and adhere to PrEP.^19^ The SEARCH study^20^ reported a decrease in HIV incidence of 74% in women who initiated PrEP. Conversely, in the recently completed vaccine trial HVTN 702,^21^ PrEP was not provided on-site and few initiated PrEP; HIV incidence was 4·2 per 100 person-years, comparable to ECHO before the introduction of PrEP.^14^, ^21^

Our study suggests that the convenience of integrated delivery of PrEP, offered on-site by study staff in the context of a clinical trial, will decrease participants' risk of acquiring HIV. Careful planning, community consultation, and staff training on PrEP initiation are important to attaining high uptake.^16^ The combined finding of modest uptake of PrEP and a substantial decrease in incidence suggest that women at higher risk, or during periods of higher risk, might also be those who choose to use PrEP. In the ECHO trial, women who initiated PrEP had characteristics correlated with increased risk, such as never married, having multiple partners, and testing positive for curable sexually transmitted infections.^16^

A limitation of our study is that access to PrEP was confounded with calendar time; thus we cannot rule out the possibility of falling HIV incidence as partly explained as a cohort effect. In addition to selecting follow-up time to reduce the potential for confounding, we evaluated trends for reducing incidence in calendar time, study time, and enrolment cohort independent of PrEP access. We found no evidence of decrease in HIV incidence during the ECHO trial that would otherwise explain the PrEP association. PrEP access was implemented in the last 8 months of the ECHO trial, and PrEP adherence is likely to be highest immediately after initiation; we were not able to address whether reduction in HIV incidence would be sustained in the longer term.

As effective PrEP approaches (eg, pills, vaginal rings,^22^, ^23^ injectables,^24^ etc) become licensed and scaled up and are increasingly accessible and acceptable, these biomedical tools will become part of the standard of prevention at the community level and in clinical trials. For trials, it could become increasingly challenging to convincingly show effectiveness of new biomedical HIV prevention technologies (eg, new PrEP drugs and HIV vaccines), if the expected rate of HIV infections will be small because of effective prevention. One strategy (being used in the MOSAICO trial; NCT03964415) is to selectively enrol participants who do not choose to initiate PrEP when offered during screening, while remaining permissive of PrEP use during follow-up. Additional statistical and operational approaches have been proposed and will need to be explored for future trials.^11^, ^25^, ^26^, ^27^

The ethical conduct of research mandates access to effective prevention, which includes PrEP. Ease of access to tenofovir disoproxil fumarate and emtricitabine PrEP has sometimes been a logistical challenge in trials, presenting a substantial barrier to actual use of PrEP. Our experience in the ECHO trial, with a substantial reduction in risk of infection coinciding with on-site access to PrEP, suggests that HIV risk can be substantially reduced by reducing barriers through integrated delivery of PrEP access for trial participants.

## Data sharing

Individual participant data including a data dictionary that underlie the results reported in this Article, after deidentification, are available for this Article. Data will be available 3 months after publication of the Article. The study protocol is available from the corresponding author. The statistical code will be available 3 months after publication from the corresponding author. Data are available for researchers who provide a methodologically sound proposal, which will be reviewed by the ECHO Management Committee. Proposals should be directed to icrc@uw.edu; to gain access, data requestors will need to sign a data access agreement and any proposal will require approval by the ECHO Management Committee.

## Declaration of interests

JMB reports grants from the Bill & Melinda Gates Foundation and United States Agency for International Development, during the conduct of the study; personal fees from Gilead Sciences, Janssen, and Merck, outside the submitted work; and since the completion of the work, he is an employee of Gilead Sciences. RH reports grants from the Bill & Melinda Gates Foundation and United States Agency for International Development, during the conduct of the study. All other authors declare no competing interests.
