# Supplementary material for: Incorporating oral PrEP into standard prevention services for South African women: a nested interrupted time-series study
Source: Lancet HIV. 2021 Jun 11;8(8):e495–501. doi: 10.1016/S2352-3018(21)00048-5 (PMC8340029; doi:10.1016/S2352-3018(21)00048-5)
Supplement: Supplementary appendix [file mmc1.pdf]

THE LANCET
HIV

Supplementary appendix

This appendix formed part of the original submission and has been peer reviewed. We post it as supplied by the authors.

Supplement to: Donnell D, Beesham I, Welch JD, et al. Incorporating oral PrEP into standard prevention services for South African women: a nested interrupted time-series study. *Lancet HIV* 2021; published online June 11. http://dx.doi.org/10.1016/S2352-3018(21)00048-5.

Supplementary Appendix

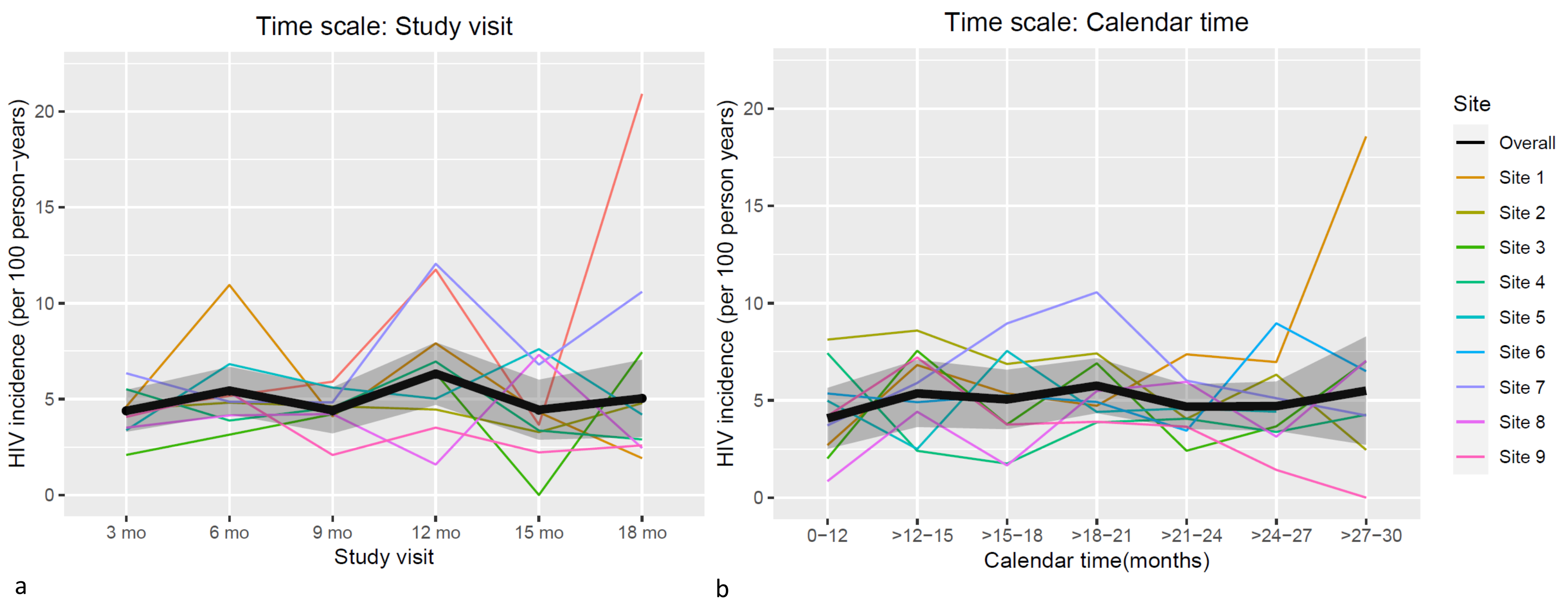

Figure S1: HIV incidence during the trial prior to on-site PrEP access

The colored lines show HIV incidence for each of the 9 South African sites; the black line shows overall HIV incidence, and the grey region the 95% confidence limit for overall incidence. Only visits prior to PrEP access are included. Two time scales are shown: a) study visit (months since participant's enrollment) and calendar time (time since first participant enrolled, in months)
